# Supplementary material for: Integrating genomic structural equation modeling and experimental validation to unravel the genetic basis of male genital lichen sclerosus
Source: Front Immunol. 2026 Jul 16;17:1857945. doi: 10.3389/fimmu.2026.1857945 (PMC13435250; doi:10.3389/fimmu.2026.1857945)
Supplement: Supplementary file 6 [file SupplementaryFile5.zip › result/fuma_PSYCH_factor_sCCA2_focus.chr20.chr20.61301855.62190180.pop1.pdf]

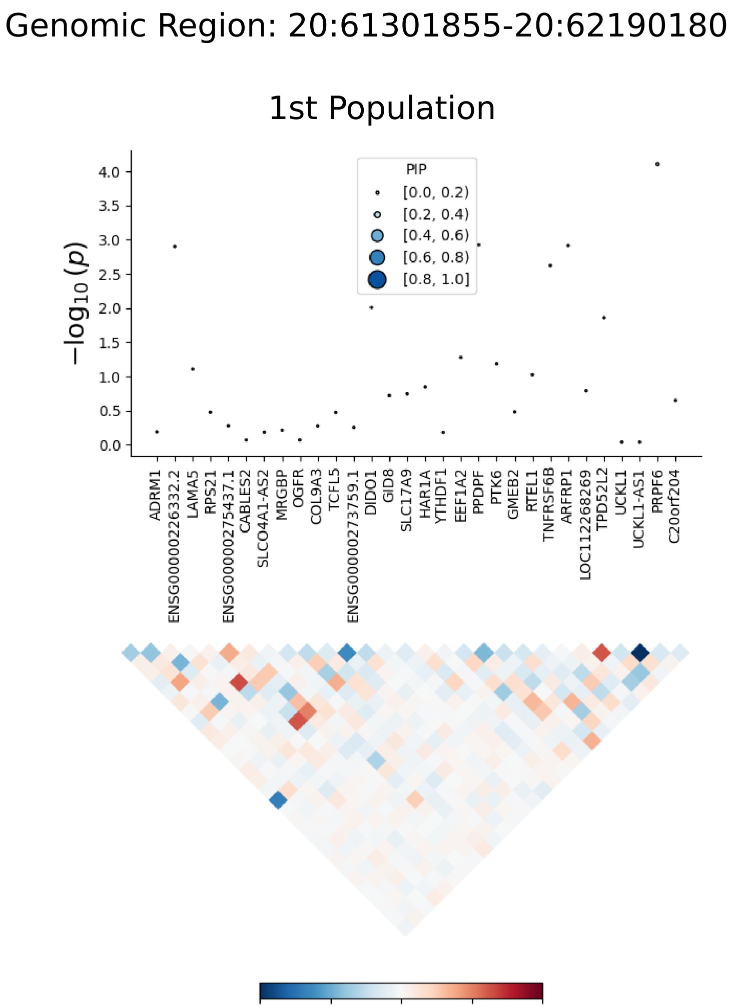
Genomic Region: 20:61301855-20:62190180
1st Population
PIP
[0.0, 0.2)
[0.2, 0.4)
[0.4, 0.6)
[0.6, 0.8)
[0.8, 1.0]
$-\log_{10}(p)$
0.0
0.5
1.0
1.5
2.0
2.5
3.0
3.5
4.0
ADRM1
ENSG00000226332.2
LAMA5
RPS21
ENSG00000275437.1
CABLES2
SLCO4A1-AS2
MRGBP
OGFR
COL9A3
TCFL5
ENSG00000273759.1
DIDO1
GID8
SLC17A9
HAR1A
YTHDF1
EEF1A2
PPDPF
PTK6
GMEB2
RTEL1
TNFRSF6B
ARFRP1
LOC112268269
TPD52L2
UCKL1
UCKL1-AS1
PRPF6
C20orf204
−1.0
−0.5
0.0
0.5
1.0
